# Characteristics of newly diagnosed COPD patients treated with triple inhaled therapy by general practitioners: a real world Italian study

**DOI:** 10.1038/s41533-017-0051-9

**Published:** 2017-09-07

**Authors:** Fabiano Di Marco, Pierachille Santus, Silvia Terraneo, Elena Peruzzi, Elisa Muscianisi, Claudio Ripellino, Valeria Pegoraro

**Affiliations:** 1 Department of Medical Sciences, Università degli Studi di Milano. Respiratory Unit, San Paolo Hospital, Milan, Italy; 2 Department of Medical Sciences, Università degli Studi di Milano. Pulmonary Rehabilitation Unit, Fondazione Salvatore Maugeri, Scientific Institute of Milan-IRCCS, Milan, Italy; 3 Epidemiology and Outcome Research Manager, Novartis Italia, Origgio, VA Italy; 4 Medical Franchise Leader Respiratory, Novartis Italia, Origgio, VA Italy; 5 Real World Evidence Consultant, QuintilesIMS, Milan, Italy

## Abstract

Factors predicting prescriptions of triple therapy were investigated in a large group of general practitioners in Italy. In the population treated by identified general practitioners, a cohort of newly diagnosed chronic obstructive pulmonary disease patients was extracted from IMS Health Longitudinal Database during the period 2010–2013. From the diagnosis, 1-year follow-up was evaluated. Thirty-two thousand forty-six newly diagnosed chronic obstructive pulmonary disease patients were evaluated (57.7% male, mean age 67 years). During 2 years prior to diagnosis less than 13% of patients were requested with a pulmonology evaluation and less than 5% with a spirometry; 65.1% cases were prescribed with a respiratory drug, which in 9.6% of cases was inhaled corticosteroid/long-acting β_2_-agonist fixed-dose combination. Two thousand and twenty eight patients (6.3% of the newly diagnosed chronic obstructive pulmonary disease patients) were treated with triple therapy during the first year of follow-up, whose 858 (42.3%) starting immediately, and 762 (37.6%) following an initial treatment with inhaled corticosteroid/long-acting β_2_-agonist fixed-dose combination. Being older, being requested with pulmonologist evaluation or spirometry, being prescribed with a inhaled corticosteroid/long-acting β_2_-agonist fixed-dose combination at diagnosis resulted independent predictors of triple therapy use.

## Chronic lung disease: ensuring correct prescriptions for early-stage disease

An improved education program for doctors promoting correct use of medication for chronic lung disease is needed in Italy. Current guidelines state that inhaled corticosteroids (ICSs) should be reserved for patients with severe chronic obstructive pulmonary disease (COPD), but it appears that doctors do not always follow this advice. Fabiano Di Marco, at San Paolo Hospital—Università degli Studi di Milano, and co-workers analyzed data from 32,046 COPD patients newly-diagnosed by family doctors in Italy between 2010 and 2013. When the researchers followed up on patients after 1 year, 2028 (6.3%) of newly-diagnosed patients were being treated with triple inhaled therapy incorporating ICSs—42% of these patients had started triple therapy immediately upon diagnosis. Being an older male and having been prescribed with a ICS/LABA FDC at diagnosis were strong predictors of triple therapy use within 1 year from the diagnosis.

## Introduction

Chronic obstructive pulmonary disease (COPD) is a common preventable and treatable disease, characterized by persistent airflow limitation that is usually progressive and associated with an enhanced chronic inflammatory response to noxious particles or gases in the airways and lungs. Exacerbations and comorbidities contribute to the overall severity of patients.^1, 2^ The destruction of lung parenchyma, increased by subtended inflammatory processes, leads to loss of alveolar attachments to the small airways and decreases lung elastic recoil; in turn, these changes diminish the ability of the airways to remain opened during expiration.^3^ COPD management is a major healthcare problem, and numerous recommendations/guidelines were created to increase appropriateness hence to address the unmet need of patients remaining symptomatic, so to improve patients’ benefit and reduce exacerbations risk.^3, 4^ International recommendations, such as the GOLD document, and national guidelines such as the ones provided by Agenas (Italian National Agency for Regional Health Care Services), provide guidance to physicians in treating COPD.^4^ Once COPD is diagnosed and its severity is established, pharmacological treatment aims primarily to improve dyspnea and quality of life, to prevent disease progression and exacerbations, and to reduce mortality.^5^ Inhaled long-acting bronchodilator therapy consists of long-acting β_2_ agonists (LABAs), and/or long-acting muscarinic antagonists (LAMAs).^6–9^ The choice within each pharmacological class relies on the availability and cost of the medication, and on the patient’s response, strictly linked to severity of symptoms, airflow limitation and rate and severity of exacerbations.^3^ Inhaled corticosteroids (ICS) are usually added as anti-inflammatory agents in case of exacerbation,^10–13^ but, in the fixed association with LABA, they have recently been demonstrated as less effective in moderate-severe patients if compared to LAMA/LABA association in terms of prevention of exacerbations.^14^ The current GOLD strategy document recommends that the use of ICS is reserved for patients with severe or very severe airflow limitation and/or ≥2 exacerbations per year (GOLD groups C and D).^5^ This recommendation is based on data from available studies, which indicated that patients with a history of frequent exacerbations were more likely to benefit from ICS treatment. Provided the availability of new compounds, during latter years, the pharmacological treatment of COPD moved toward a more personalized approach, such as targeting the eosinophils, the asthma and COPD overlap syndrome or the so-called phenotype of the frequent exacerbator,^15^ and bringing triple ICS/LABA/LAMA therapy to phase III.^16, 17^

Nevertheless, despite GOLD recommendations and even more recent evidences underlining the efficacy of LABA/LAMA FDC vs. ICS/LABA fix dose combination (FDC),^14, 18^ and despite a clear identikit of severe COPD patient possibly benefitting from adequate triple therapy with ICS/LABA/LAMA, ICS and ICS/LABA FDCs are often prescribed in the early stage of the disease.^19, 20^ Even if national recommendation in Italy suggest general practitioner (GP)/specialist interaction, currently GPs are allowed to manage both the diagnosis (requesting spirometry or, in limited cases, performing spirometry themselves), and the treatment of COPD, with the only exception of the prescription of LABA/LAMA FDCs, and roflumilast which need currently specialists’ indication.

The aim of the present study was to describe newly diagnosed COPD patients according to their characteristics observed during 2 years prior to diagnosis and to identify factors that could predict progression to triple therapy in the first year after diagnosis in a real-life context of GPs setting in Italy.

## Results

From the analysis of IMS Health LPD database, 32,046 newly diagnosed COPD patients have been found, whose 2028 (6.3%) have been treated with triple inhaled therapy in the year of follow-up.

### Feature of overall population

Characteristics of the overall cohort are shown in Table 1. The majority of patients were male, with a mean age of 67 years, and the majority of them expressing comorbidities (94.0%): as expected, a high rate of cardiovascular diseases (65.0%) and diabetes (16.8%), but also anxiety, and depression were found (6.3 and 5.8%, respectively). The registration of smoking history was low, since in more than one out of four patients this information was not collected. Moreover, more than one out of four patients presented with obesity (i.e., BMI ≥ 30 Kg/m^2^, Table 1, information registered in 76.4% of cases). In the 2 years before diagnosis (i.e., pre-selection period), less than 13.0% of patients were requested with a pulmonology evaluation and less than 5% of the patients were requested with a spirometry. It is noteworthy that the same approach was used also for patients treated with at least one respiratory drug in the 2 years prior to diagnosis (65.1%), since more than 80.0% were not requested a specialist evaluation nor a spirometry (Table 1). Three-thousand sixty-nine patients have been treated during the 2-year period before diagnosis with ICS/LABA FDC (9.6% of the overall population, and 14.7% of patients prescribed with any therapy, Table 1).

**Table 1:** Features of newly diagnosed COPD patients (overall population *n* = 32,046)

Feature	
Age, mean ± SD, years	67 ± 15
<40 years, *n* (%)	1452 (4.5%)
≥80 years, *n* (%)	7199 (22.5%)
Female, *n* (%)	13558 (42.3%)
BMI, mean ± SD, Kg/m^2^	27.5 ± 5.3
BMI < 18.5, *n* (%)	510 (2.4%)
BMI ≥ 30, *n* (%)	5853 (27.5%)
Smoking habit missing, *n* (%)	8832 (27.6%)
Current smokers, *n* (%)	8004 (25.0%)
Comorbidities	
None, *n* (%)	1940 (6.0%)
Cardiovascular disease, *n* (%)	20834 (65.0%)
Diabetes, *n* (%)	5397 (16.8%)
Osteoporosis, *n* (%)	3653 (11.4%)
Anxiety, *n* (%)	2009 (6.3%)
Depression, *n* (%)	1851 (5.8%)
Anemia, *n* (%)	908 (2.8%)
Reported respiratory symptoms^1^, *n* (%)	11,885 (37.1%)
Hospitalization in respiratory ward^1^, *n* (%)	248 (0.8%)
Pulmonology visit request^1^, *n* (%)	4149 (12.9%)
With spirometry, *n* (%)	2538 (61.2%)
Spirometry request^1^, *n* (%)	1473 (4.6%)
Respiratory treatment (any)^1^, *n* (%)	20,859 (65.1%)
Without pulmonology visit, *n* (%)	17,250 (82.7%)
Without spirometry, *n* (%)	17,515 (84.0%)
Respiratory treatment with ICS/LABA FDC^1^, *n* (%)	3069 (9.6%)

*ICS/LABA FDC* inhaled corticosteroid/long-acting β_2_ agonist fixed-dose combination, *SD* standard deviation

^1^ In the pre-selection period (i.e., 2 years before the diagnosis of COPD)

### Features of patients treated with triple therapy

At the end of the first year of follow-up period, 2028 patients (6.3% of the newly diagnosed COPD patients) were treated with triple therapy, whose 858 (42.3%) starting immediately at the time of diagnosis, while 762 (37.6%) following an initial treatment with ICS/LABA FDC. Mean time to triple therapy since first COPD diagnosis was estimated in 61 ± 91 days, with median time of 14 days.

Results from the comparison between patients initiated to triple therapy within 1 year since the first diagnosis and those who did not are shown in Table 2. Patients prescribed with triple therapy resulted on average 5 years older, more frequently male, and affected by one or more comorbidities. As described in Table 2, COPD symptoms frequency varied a lot accordingly with being prescribed with triple therapy, with 56.8% and 35.7% of patients with at least one COPD symptoms in the subgroup of patients that went to triple therapy, and in the subgroup of patients who didnot, respectively (*P* < 0.001, Table 2). In the 2 years before diagnosis, the approach of the GPs resulted different in the two subgroups, since more patients initiated to triple therapy had at least one pulmonologist visit, one spirometry, or have been treated with a respiratory treatment (*P* < 0.001 for all comparisons, Table 2). Also, the proportions of patients who had at least one ICS/LABA FDC prescription at COPD diagnosis resulted very different: among patients prescribed with triple therapy, 762 (37.6%) had at least one ICS/LABA FDC prescription in comparison with 7.7% among those who were not prescribed with triple therapy (*P* < 0.001).

**Table 2:** Comparison between patients prescribed or not with triple therapy

	Treated with triple therapy (*n* = 2028)	Not treated with triple therapy (*n* = 30018)	*P*
Age, mean ± SD, years	72 ± 11	67 ± 15	<0.001
Female*, n* (%)	789 (38.9%)	12769 (42.5%)	0.001
BMI, mean ± SD, Kg/m^2^	27.9 ± 5.5	27.4 ± 5.3	0.013
Current smokers*, n* (%)	372 (25.9%)	7632 (35.0%)	<0.001
Comorbidities^1^ *, n* (%)	1555 (76.7%)	21365 (71.2%)	<0.001
None*, n* (%)	1779 (2.9%)	161(3.7%)	0.002
CV Diseases*, n* (%)	19,394 (31.4%)	1440 (33.1%)	0.015
Depression*, n* (%)	1716 (2.8%)	135 (3.1%)	0.200
Osteoporosis*, n* (%)	3412(5.5%)	241(5.5%)	0.938
Anemia*, n* (%)	855(1.4%)	53 (1.2)	0.371
Diabetes*, n* (%)	5024(8.1%)	373 (8.6%)	0.286
Anxiety states*, n* (%)	1893(3.0%)	116 (2.7%)	0.145
COPD symptoms^2^ *, n* (%)	1153 (56.6%)	10732 (35.8%)	<0.001
Spirometry request^2^ *, n* (%)	580 (28.6%)	3431 (11.4%)	<0.001
Pulmonology visit request^2^ *, n* (%)	665 (32.8%)	3484 (11.6%)	<0.001
Respiratory treatment^2^ *, n* (%)	1592 (78.5%)	19,267 (64.2%)	<0.001
ICS/LABA FDC at diagnosis*, n* (%)	762 (37.6%)	2307 (7.8%)	<0.001

*ICS/LABA FDC* inhaled corticosteroid/long-acting β_2_ agonist fixed-dose combination, *SD* standard deviation

^1^ Patients with at least one comorbidity among the following ones: cardiovascular diseases, diabetes, osteoporosis, anxiety, depression, and anemia

^2^ In the pre-selection period (i.e. 2 years before the diagnosis of COPD)

Multivariate cox regression model showed a statistically significant association between triple therapy and all the studied covariates included in the model, with none of them excluded according to the stepwise approach adopted (Table 3). Being so, male patients, elderly patients and patients requested with a pulmonology visit or just a spirometry were more likely prescribed with triple therapy than other patients. Moreover, patients who presented COPD symptoms during the pre-selection period were more likely prescribed with triple therapy, whereas comorbidities presence had the opposite effect. Furthermore, being prescribed with a ICS/LABA FDC at the first COPD diagnosis seemed to be a strong predictor of being prescribed with triple therapy within 1 year since diagnosis, with a HR of 5.10 (95% CI: 4.65–5.60, Table 3).

**Table 3:** Multivariate cox regression model to predict prescription of triple therapy

	Hazard ratio	95% Confidence interval
Female (vs. male)	0.81	0.74–0.89
Age classes		
<40	1.00	
40–50	2.99	1.58–5.67
50–60	4.70	2.56–8.65
60–70	7.57	4.16–13.79
70–80	9.77	5.37–17.79
≥80	9.86	5.41–17.98
COPD symptoms^1^	1.54	1.40–1.70
Comorbidities^2^	0.80	0.72–0.90
Pulmonology visit request^1^	1.98	1.76–2.24
Spirometry request^1^	1.26	1.12–1.43
ICS/LABA FDC at diagnosis	5.10	4.65–5.60

*ICS/LABA FDC* inhaled corticosteroid/long-acting β_2_ agonist fixed-dose combination

^1^ In the pre-selection period (i.e., 2 years before the diagnosis of COPD)

^2^ Patients with at least one comorbidity among the following ones: cardiovascular diseases, diabetes, osteoporosis, anxiety, depression, and anemia

## Discussion

### Main findings

In this large GPs setting, following results were found: (1) symptomatic, males, older patients, patients with less comorbidities and patients prescribed with a pulmonology visit or just a spirometry were more likely prescribed with triple therapy; (2) prescription of ICS/LABA FDC at the time of diagnosis and older age seemed to be the strongest predictors for triple therapy use. (3) in the 2 years before the diagnosis few patients were requested with a pulmonology evaluation or a spirometry, even if treated with a respiratory drugs.

### Interpretation of findings in relation to previously published work

Our newly diagnosed COPD patients’ cohort presents demographic characteristics similar to those found in a cohort of patients collected in an analog real world evidence study conducted in UK,^21^ with both studies describing a mean age of about 67 years and a mean BMI of 27 kg/m^2^ for patients at their first COPD diagnosis. Results in terms of comorbidities are in line with those found by the INDACO study,^22^ reporting a high prevalence of comorbidities among COPD patients, with more than 80% presenting with at least one comorbidity. Patients with recorded symptoms before being diagnosed with COPD represented 37%. Considering that symptomatology recorded in the database is not mandatory unless leading to a prescription, specialist visit or exam request, this proportion should be regarded as quite high. In addition, it should be kept in mind that the analyzed setting is the one of GPs. Pulmonologist visit including FEV1 evaluation were found just in a small group of patients. Pulmonologists visits and FEV1 evaluations could increase the probability of a better management as well as to a reduced delay in proper diagnosis. In addition patients that had at least one respiratory treatment before diagnosis, represented 65.1% of the total population. Delay in COPD diagnosis by GPs has been confirmed also in previous studies. When comparing results about the proportion of patients prescribed with ICS/LABA FDC at the time of the first COPD diagnosis, results from the present study showed a approximate 10% lower proportion than the one found in the above mentioned UK study (approximately 43%).^21^ It should be taken in account that for the UK study is not specified whether LABA and ICS combinations are fixed, extemporaneous or both, while in the present study only fixed combinations have been evaluated. Nevertheless, when evaluating the proportion of patients that within 1 year progress to triple therapy among those prescribed with ICS/LABA FDC at the time of first diagnosis, the present study revealed a higher percentage (25% coming from 762 patients over 3069 prescribed with ICS/LABA FDC vs. 17% from UK study). Proportions become more similar when comparing the 25% of patients going to triple therapy within 1 year (Italy), with the 24% of patients going to triple therapy within 2 years (UK), leading to the consideration that, probably, Italian GPs are more cautious at prescribing ICS/LBA FDC as a first line therapy, but at the same time the step up in therapy happens faster when compared to the English setting. The rapidity Italian GPs escalate therapy as observed in the present study, could be related to the fact that ICS/LABA are prescribed as fixed dose combinations.

The reduced likelihood of being prescribed with triple therapy observed for patients affected by other comorbidities is an interesting finding that should be contextualized in the GPs’ setting: research and guidelines on the management of long-term conditions have routinely focused on single diseases,^23^ while patients with comorbidities are usually excluded from randomized controlled trials.^24^ This has led to individual disease management rather than a more holistic approach that, on the other hand, should be the approach adopted by GPs. The more chronic conditions a patient has, the more medications they are likely to be prescribed.^25, 26^ Multiple therapies commonly lead to drug disease interaction and drug–drug interactions:^17, 27, 28^ these issues, as well as the fear of adverse drug reactions, are the factors probably preventing GPs’ from prescribing triple therapy to patients affected by comorbidities, possibly due to the conception of COPD as less important than other chronic disease (e.g., cardiovascular disease or diabetes). However, drugs for COPD are inhaled, thus much less likely to cause systemic adverse effects and drug interactions than systemic ones.

### Strengths and limitations of this study

The large number of patients included in the analysis has to be considered among the study strengths’, together with the longitudinal nature of data collected, the generalizability of Italian general practice population, and the usage of validated codes for identifying COPD patients. Among study limitations, it should be addressed that smoking habits information were not considered since in the database data about this issue are not complete. These data confirm a study by Bryant et al.^29^ describing that accurate detection of smoking habits occurs in less than two-thirds of all patients, despite significant investment to increase GPs’ intervention for lifestyle risk factors, even if the possibility that GPs discussed this aspect with their patients without including it in the medical records cannot be ruled out. Moreover, this kind of information in a real practice context could not be reliable due to the fact that, being based on what patients report to the GPs, they are self-reported measures, and it is known that self-reported information about smoking status are biased.^30^ The study was also limited by the lack of available information on FEV_1_ values, as well as hospitalizations and exacerbations, which would have provided a more comprehensive picture of COPD patients’ management and would have allowed a stratification by disease severity. Nevertheless, results from a previous study using the same source of data, where FEV_1_ was recorded to define disease severity as per GOLD guidelines, suggest that the presence of FEV_1_ value does not constitute a bias: patients resulted to be distributed among disease severity classes, with mild and moderate patients accounting for about 80% of the cohort.^31^ Another limitation is the lack of spirometry assessment to diagnose COPD: this approach is common for all “real life” studies, currently proposed also to evaluate the effectiveness of new treatments (Salford study). This bias, which should not affect the interpretation of the results, is in line with the aim to evaluate the behavior of GPs in real clinical practice. Then, “Chronic bronchitis”, a condition different from COPD in the absence of obstruction, was also included in the analysis, given the term often used as a synonymous for COPD. Finally, the role of GPs is different in several countries, condition which limits the external validity of our study.

### Implications for future research, policy, and practice

Even if there is an Italian document/guideline on COPD diagnosis and treatment, the results of our study failed to demonstrate an appropriate management of patients^32^ suffering from this disease. In this respect, new campaigns of information are desirable: following such an informative approach, a new study to evaluate whether the approach to COPD has improved is needed, mainly in terms of early diagnosis with spirometry, and appropriate treatment and follow-up. Then, accessibility of spirometry in primary care has to be facilitated.

### Conclusions

In the studied population, the choice of treatment is driven by a set of factors that include symptoms, general patients’ features as well as functional and specialists evaluation. However, the proportions of patients with a request for a specialist evaluation or a spirometry are still very low. Even if the last version of GOLD document gives a high level of importance to symptoms for the choice of the treatment, the same document underline the importance of spirometry to confirm the diagnosis and to assess the level of the flow limitation. In this setting educational programs should be implemented for GPs in order to optimize the diagnosis treatment and follow up of patients with COPD.

## Methods

### Data source

The data used in the present study were retrieved from the Italian General Practitioners’ (GPs) IMS Health Longitudinal Patient Database (IMS Health LPD) established in 1998 primarily to carry out epidemiologic studies and to monitor drugs prescriptions and drug safety. The database is fed by a constant representative doctors’ sample that agreed to take part to a research panel, has been trained for data entry and uses standard patient management software to collect data directly during the consultations. The information, which is gathered continually and in real time allows patients and doctors to be longitudinally monitored and includes anonymous data about patients’ demographics, medical diagnoses, drug prescriptions, hospital referrals, diagnostic investigations, and date of death. Medical diagnoses and drugs prescriptions, both coded directly by GPs, comply with the ninth edition of International Classification of Disease (ICD-9-CM), and the Anatomical Therapeutic and Chemical classification system, respectively. All medical records are linked together with a unique encrypted patient code. In Italy, IMS Health LPD consists of a panel of about 900 GPs homogenously distributed throughout the national territory; it collects information about 1 million patients per year and it is representative of the Italian general population. IMS Health LPD database reliability has been recognized by the pharmaceutical industry and medicine agencies.^33^

### Ethics

The study was performed in accordance with Good Epidemiological Practice guidelines. Ethics board approval was not required because the data were not nominative and were collected retrospectively.

### Cohort selection

Newly diagnosed COPD Cohort was selected in the period from 1 January 2010 to 31 December 2013, as patients with a COPD diagnosis, defined as “Chronic bronchitis”, “Emphysema” or “Chronic airway obstruction, not elsewhere classified”. The presence of at least one registration within the database of one of the above-mentioned ICD-9-CM codes was the only criteria to define COPD diagnoses (i.e., not necessarily confirmed by spirometry). In order to identify the first diagnosis, patients with a diagnosis during the previous 3-year period were excluded from the analysis. The date of the first COPD diagnosis (i.e., index date) during the selection period was set as the 1 years follow-up period start.

### Variables analyzed during the pre-selection period

For each patient, information about COPD symptoms (see Appendix), spirometry execution, respiratory treatments (see Appendix), comorbidities (defined by means of ICD-9 codes registrations), and pulmonologist visits requests over a 2-year period before the first COPD diagnosis (i.e., pre-selection period) were collected and analyzed to characterize patients and used as covariate in the analysis.

### Follow-up and outcomes

Each cohort member was followed from the date of first COPD diagnosis until outcome onset (see below), censoring, i.e., death, emigration, or end of follow-up (i.e., 12 months after the date of first COPD diagnosis), whichever date occurred earlier. Main outcome (i.e., triple therapy presence during follow-up) was defined as the presence of concomitant prescriptions of LABA, LAMA, and ICS. Prescriptions were considered concomitant when the lapse of time between them was less than 30 days. Time to triple therapy was calculated as the difference, in days, between the date of the first prescription that defined a triple therapy presence during the follow up period, and the first COPD diagnosis date.

### Statistical analysis

Newly diagnosed COPD patients were described in terms of demographic, and clinical characteristics; qualitative variables were described using usual statistical methodologies, which are frequencies and percentages, whereas quantitative variables were described in terms of mean ± standard deviation. Chi-square test was calculated to compare patients who received triple therapy within 1 year since the first COPD diagnosis to patients who did not. Comparison was based on presence/absence of respiratory treatments, spirometry execution, pulmonologist visit requests, and COPD symptoms during the pre-selection period and presence/absence of at least one LABA/ICS FDC prescription at the first COPD diagnosis. To evaluate the potential association between triple therapy and factors that could influence the likelihood of being prescribed triple, age, sex, presence/absence respiratory symptoms before diagnosis, comorbidities, pulmonology visit, spirometry, and prescriptions LABA/ICS FDC at the first COPD diagnosis were considered in both univariate and multivariate Cox regression models. For multivariate models, each variable had been retained in the final model according to a stepwise approach. A *p*-value below than 0.15 was the cutoff. For all the above-mentioned analysis, *p*-values below than 0.05 had been considered as statistically significant.

### Data availability

The data that support the findings of this study are available from QuintilesIMS, but restrictions apply to the availability of these data, which were used under license for the current study, and so are not publicly available. Data are, however, available from the corresponding author upon reasonable request and with permission of QuintilesIMS.

## Electronic supplementary material

Appendix 1. Diagnosis, symptoms and respiratory treatments included in the study
