# Supplementary material for: Characteristics of newly diagnosed COPD patients treated with triple inhaled therapy by general practitioners: a real world Italian study
Source: NPJ Prim Care Respir Med. 2017 Sep 7;27:51. doi: 10.1038/s41533-017-0051-9 (PMC5589801; doi:10.1038/s41533-017-0051-9)
Supplement: Supplementary file 1 — Appendix 1. Diagnosis, symptoms and respiratory treatments included in the study [file 41533_2017_51_MOESM1_ESM.docx]

Appendix 1. Diagnosis, symptoms and respiratory treatments included in the study.

| **ICD-9-CM Codes used to define COPD diagnosis** |
| --- |
| 491: Chronic bronchitis  492: Emphysema  496: Chronic airway obstruction, not elsewhere classified |
| **ICD-9-CM Codes used to define COPD symptoms** |
| 466.0: Acute Bronchitis  786.2: Cough  490: Bronchitis  493: Asthma  786.0 Dyspnea  518: Other Diseases of Lung  786.7: Abnormal Chest Sounds  786.4: Abnormal Sputum |
| **ATC Codes used to define respiratory treatments** |
| *Short-Acting β2 Agonist (SABA)*  Fenoterol: R03AC04  Levalbuterol: R03AC02  Salbutamol: R03AC02, R03CC02  Terbutaline: R03AC03, R03CC03  *Long-Acting β2 Agonist (LABA)*  Formoterol: R03AC13  Indacaterol: R03AC18  Salmeterol: R03AC12  *Short-Acting Muscarininc Antagonist (SAMA)*  Ipratropium bromide: R01AX03, R03BB01  Oxitropium bromide: R03BB02  *Long-Acting Muscarininc Antagonist (LAMA)*  Aclidinium bromide: R03BB05  Glycopyrronium bromide: R03BB06  Tiotropium: R03BB04  Umeclidinium: R03BB07  *Fixed-dose combination (FDC) SABA + SAMA*  Fenoterol/ipratropium: R03AL01  Salbutamol/ipratropium: R03AL02  *Methylxanthines*  Aminophylline: R03DA05  Theophylline (SR): R03DA04  *Inhaled corticosteroids (ICS)*  Beclomethasone: R03BA01  Budesonide: R03BA02  Fluticasone: R03BA05  *FDC: LABA + Corticosteroids*  Formoterol/budesonide: R03AK07  Formoterol/beclometasone: R03AK08  Formoterol/mometasone: R03AK09  Salmeterol/fluticasone: R03AK06  Vilanterolo/fluticasone: R03AK10  *FDC: LABA + LAMA*  Indacaterolo/glicopirronio: R03AL04  Vilanterol/Umeclidinium: R03AL03  *Systemic corticosteroids*  Prednisolone: H02AB06  methyl-prednisolone: H02AB04  *Phosphodiesterase-4 inhibitors*  Roflumilast: R03DX07  *Tetracycline:* J01A  *II generation oral cephalosporin:* J01DC  *Macrolides*  Claritomicine: J01FA09  Azitromicine: J01FA10  *Fluorochinolonici*  Moxifloxacine: J01MA14  Levofloxacin: J01MA12  Ciprofloxacin: J01MA02  *Amoxicillin*  Amoxicillin without clavulanato: J01CA04  Amoxicillin with clavulanato: J01CR02  *Cefotaxime:* J01DD01  *Ceftriaxone:* J01DD04  *Ceftazimide:* J01DD02  *Cefipime:* J01DE01 |
